# Effects of LED Light Color and Intensity on Feather Pecking and Fear Responses of Layer Breeders in Natural Mating Colony Cages

**DOI:** 10.3390/ani9100814

**Published:** 2019-10-16

**Authors:** Haipeng Shi, Baoming Li, Qin Tong, Weichao Zheng, Dan Zeng, Guobin Feng

**Affiliations:** 1 Department of Agricultural Structure and Bioenvironmental Engineering, College of Water Resources & Civil Engineering, China Agricultural University, Beijing 100083, China; shihaipeng@cau.edu.cn (H.S.); libm@cau.edu.cn (B.L.); tongqin@cau.edu.cn (Q.T.); 2 Key Laboratory of Agricultural Engineering in Structure and Environment, Ministry of Agriculture and Rural affairs, Beijing 100083, China; 3 Beijing Engineering Research Center on Animal Healthy Environment, Beijing 100083, China; 4 Hebei Industrial Technology Research Institute of Layers, Handan 056800, China; 15101124340@163.com (D.Z.); feng181020@sina.com (G.F.)

**Keywords:** poultry, light spectrum, light illuminance, feather pecking, cannibalism, animal welfare

## Abstract

**Simple Summary:**

Commercial breeder farms are moving forward using colony cages due to high efficiency, low energy input, clean production, and as a result of the rising public concerns with respect to the welfare of hens in conventional cages. Compared with conventional cages, layer breeders in colony cage are the parent-stock of laying hens and are confined together with roosters. However, the use of colony cages is still in a preliminary stage due to behavioral issues such as feather pecking (FP) and cannibalism. These behaviors can cause poor health, poor welfare, and economic problems. It is necessary to identify effective and proximal management practices to alleviate the damage that is caused by FP and cannibalism in such colony cage systems. This study aims to mitigate the problems of FP and cannibalism by utilizing light environment regulation. Results of this study indicates that red light and low light intensity could effectively alleviate FP and cannibalism during the laying period. Such knowledge might help to understand FP behavior and stress susceptibility of hens in this system and will provide a basis for the optimization of the cage equipment and the regulation of light environment.

**Abstract:**

Natural mating colony cages for layer breeders have become commonplace for layer breeders in China. However, feather pecking (FP) and cannibalism are prominent in this system. The objective of this study was to investigate the effects of four light-emitting diode (LED) light colors (white: WL, red: RL, yellow-orange: YO, blue-green: BG) with two light intensities for each color, on FP, plumage condition, cannibalism, fear, and stress. A total of 32 identical cages were used for the eight treatments (four replicates for each treatment). For both light intensities, hens in RL had a lowest frequency of severe FP, whereas hens in WL had the highest frequency of severe FP. Hens in RL and BG had better plumage conditions than in WL and YO. Compared with RL and BG treatments, hens treated with WL and YO had a significantly longer tonic immobility (TI) duration. Hens treated with RL had a higher concentration of 5-hydroxytryptamine (5-HT), a lower concentration of corticosterone (CORT), and a lower heterophil to lymphocyte ratio than WL and YO. Furthermore, RL could significantly reduce mortality from cannibalism. Overall, hens treated with RL and low light intensity showed a lower frequency of severe FP, less damaged plumage, were less fearful, had lower physiological indicators of stress, and had reduced mortality from cannibalism. Transforming the light color to red or dimming the light could be regarded as an effective method to reduce the risk of FP and alleviate the fear responses of layer breeders.

## 1. Introduction

The rising public concern for poultry welfare and increasing labor costs have resulted in in stacked natural mating colony cages becoming a trend in housing systems for commercial layer breeders in China. Layer breeders are the parent-stock of laying hens and in colony cages are confined together with roosters. The ratio of roosters and hens is generally kept between 1:10 and 1:8 and the flock size is usually maintained between 40 and 100 per individual cage. Compared with the cage system using artificial insemination, the natural mating behavior of breeding hens can be expressed in the natural mating colony cage system, taking into account animal welfare, high efficiency, energy savings, and clean production characteristics [1]. However, this housing system is still in the stage of exploration and optimization. Behavioral issues such as feather pecking (FP) and cannibalism are prominent in this system, contributing to economic losses and diminished health and welfare of hens. Currently, limited systematic research on FP and cannibalism in natural mating colony cages can be found. Available and efficient management measures are urgently required to ease the negative effects caused by FP and cannibalism in this colony cage system.

Feather pecking and cannibalism can occur as a result of numerous factors including genetic background [2], hormones [3], nutrition [4], group size and stocking density [5], and environmental enrichment [4]. Indeed, light management is a crucial eliciting factor of the incidence and severity of FP and cannibalism of hens [6]. Measures such as keeping the hens under a reduced light intensity or altered light color are usually adopted to alleviate FP and cannibalism when necessary [6,7]. The objective of dimming the light or altering the light color is to diminish the birds’ perception of colors and visual detection among them [8].

Excessive light is a vital factor initiating and favoring FP and cannibalism [9]. It was reported by Blokhuis and Arkes [10] that higher light intensity strongly impacts the occurrence and severity of FP in hens, resulting in more pecking damage. Reduced feather pecking behaviors and incidence of aggressive behaviors were observed by lowering the light intensity according to the results of Braastaad [6]. Hens confined close to light sources at an intensity level of 11–44 lux were more likely to perform FP than those further away where the light intensity ranged from 1 to 11 lux [11]. However, Kjaer and Sørensen [12] found that light intensity had no impact on the frequency of FP in any of the tested genotypes. Experimental results on the effects of light color on FP or aggression behavior are contradictory [7,13,14]. Due to other environmental effects and the strain differences between hens, it is difficult to draw any firm conclusions from these experiments.

Light-emitting diodes (LEDs) are a special kind of semiconductor diode which can give monochromatic light. Compared to incandescent light and fluorescent light, LED light has a marked longer life, specific spectrum, lower thermal output, higher energy efficiency, and higher reliability and frequency, as well as lower maintenance costs [7,13,15]. Knowledge about the influence of light condition on FP behavior is well documented for laying hens in other housing systems and strains, such as Oakham Blue [8], White Leghorn [12], and Brown Nick laying hens [16] in free-range systems, ISA Brown [9] and Lohmann Brown [17] hens in deep litter systems, Dekalb white breed hens in aviary systems [18], White Leghorns hens in battery cages [19], and so on. However, effects of LED light wavelength and intensity on FP and cannibalism have rarely been investigated in natural mating colony cages. The results of both wavelength and light intensity on the behaviors of laying hens in other housing systems may not be applicable to this colony cage system. Therefore, it is crucial to explore the effects of LED light color and intensity on FP and cannibalism in order to provide a basis for the regulation of light environment for layer breeders in natural mating colony cages. The objectives of this study were to investigate the effects of four LED light colors (white, red, yellow-orange, blue-green) with two light intensities in each color on FP, plumage condition, mortality from cannibalism, fear, and stress hormones for layer breeders in natural mating colony cages.

## 2. Materials and Methods

### 2.1. Animals and Experimental Treatments

**Figure 1:**
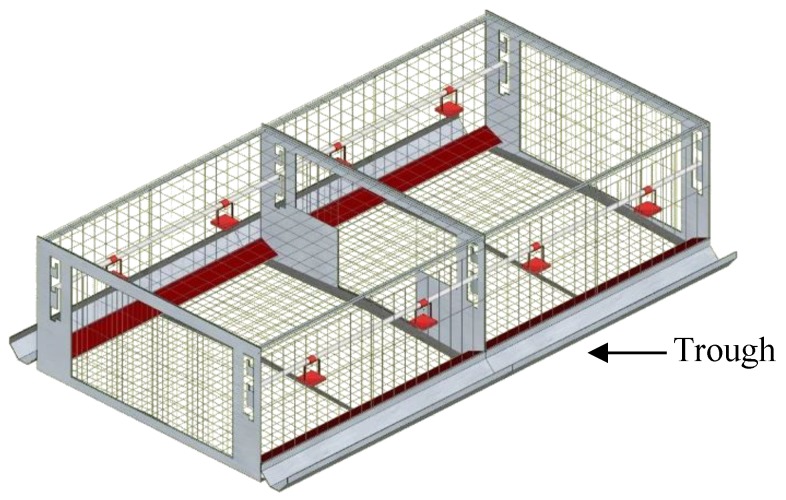
Schematic diagram of the natural mating colony cage.

All birds were managed by trained staff and the procedures relating to the use of live birds in this experiment were approved by The Laboratory Animal Ethical Committee of China Agricultural University (2018-0038). The experiment was carried out in Huayu Poultry Breeding farm, which is located in Handan, Hebei province, China. Hy-Line Brown parent-stock pullets (*n* = 1440) and cockerels (*n* = 160) were obtained from a commercial breeder and transferred into the experimental house at the age of 16 weeks and randomly distributed into 32 identical natural mating colony cages (2.40 × 1.20 × 0.71 m, length × width × height) with five males and 45 females in each cage. All double-sided experimental cages were arranged in four rows of two tiers. Each experimental cage had a floor area of 2.88 m^2^, equipped with commercial feed and drinking facilities (Figure 1). The feed was evenly distributed in the trough and automatically distributed four times a day at 07:00, 11:00, 15:00, and 19:00 h to ensure birds had permanent ad libitum access to feed. All birds were provided the same stand diet, containing (g/kg; calculated) 178 CP, 4.2 Met, 8.5 Lys, 38.2 Ca, 6.5 Pt, and 11.4 MJ ME/kg. Eggs and manure were collected once a day through egg conveyor belts and manure belts, respectively. Average air temperature and relative humidity were maintained between 16 °C and 23 °C, at 50% and 80%, respectively. All birds in the house were reared following the standard guidelines for Hy-Line Brown layer breeders of Hebei Huayu Poultry Breeding Co. Ltd. (Handan, Hebei, China).

**Figure 2:**
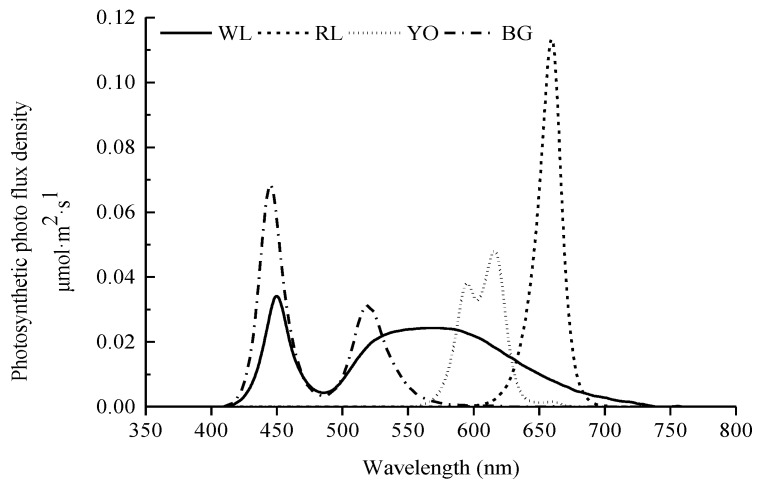
Light spectral distribution of four light-emitting diode (LED) lights (WL: white, RL: red, YO: yellow-orange, BG: blue-green).

**Figure 3:**
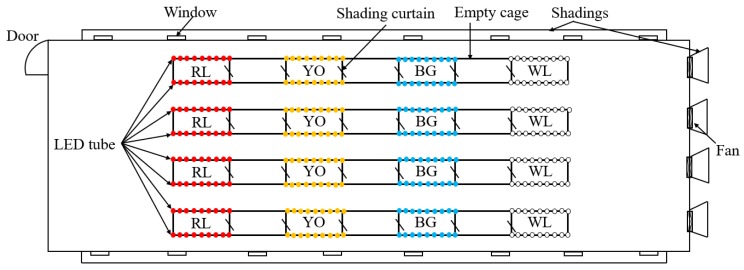
Schematic diagram of the layout of the four LED tubes (WL: white, RL: red, YO: yellow-orange, BG: blue-green).

Eight treatments were offered in this study with four LED light colors, each at two light intensities, and giving four replicate cages for each light treatment. As shown in Figure 2, the four LED light colors were (1) red LED light (RL), at a peak wavelength (λp) of 660 nm and a dominant wavelength (λd) of 641 nm, half band width (Δλd) of 20 nm; (2) yellow-orange LED light (YO), λp = 616 nm, λd = 600 nm, Δλd = 38 nm; (3) blue-green LED light (BG), λp = 445 nm, λd = 479 nm, Δλd = 21 nm; and (4) white LED light (WL), λp = 449 nm, λd = 491 nm, Δλd = 23 nm. All LED light lamps (Huazhaohong Optoelectronic Technology Co. Ltd., Wuxi, China) were installed at the upper-tier cages, which were attached to the two sides of the cage celling. For all rows, starting from one end of the house, the cages were lit with red, yellow-orange, blue-green, and white LED light, respectively (Figure 3). Voltage for red, yellow-orange, blue-green, and white LEDs was tuned based on the relative spectral sensitivity curve indicated by Prescott and Wathes [20], so that the four lightings appeared iso-illuminant to hens. Light intensity was measured at the level of birds’ heads using a precision luminometer (SRI-PL-6000, Shang Ze Photoelectric Co. Ltd., Taiwan, China) with a resolution of 0.01 lux according to human spectral sensitivity. The light intensity of the upper tier was 25 lux (high light intensity: HLI), and the lower tier was 10 lux (low light intensity: LLI). Experimental cages of different light colors were separated by an empty colony cage to avoid light pollution between different light colors. During the experiment period, the lighting rhythm was adjusted based on the different age phase, with a starter 8-h light at the age of 16 and 17 weeks and 10-h light at the age of 18 weeks, and then increased stepwisely each week to reach 16-h light at the age of 30 weeks. The samplings in each treatment were adjusted according to each measurement (Table 1). During the experiment, the same focal birds were used for samplings in each treatment.

### 2.2. Behavioral Observations

The pecking behavior of the birds in each experimental cage was recorded by direct behavior sampling for 1 h periods. For each cage, 12 focal hens from tag numbers 1 to 12 were separately observed, lasting 5 min for each of them. Observations were made by two trained people over 4 days during 34 weeks. Hens from four cages were observed by each observer in 1 day: two cages in the morning, two in the afternoon. The order of observing each treatment cage, and time of day (am and pm) were balanced in a Latin square design to guarantee inter-observer agreement on behavior recording, the two observers developed proficiency in use of the ethogram before commencing formal data collection. Observation principles were brought into correspondence with each other and frequent checks were made for the consistency of inter-observer reliability during data collection. Frequencies of severe FP (SFP, forceful pecks, sometimes with feathers being pulled out, with the recipient bird moving away), gentle FP (GFP, slow and calm pecks, not resulting in feathers being pulled out, usually without reaction from the recipient bird), aggressive pecking (SP, fast and singular pecks, mainly directed at the head or other parts of the facial region), environmental pecking (ENP, pecks at the floor and other objects in the cage), and food pecking (FOP, pecks at the feeder and drinker) were recorded on a prepared check-sheet. A new bout of pecking behavior was recorded when there had been an interval 4 s or more between two feather pecks. Throughout this experiment, hens with bleeding wounds caused by injurious pecks by other conspecifics in all experimental cages were recorded. Injurious pecks targeted to cloacal, cannibalism of feathered body parts were separated. Number of dead birds led by cannibalism and other casualties was also recorded.

### 2.3. Fear Tests

#### 2.3.1. Open Field Test

Six focal hens from each experimental cage were tested individually for their responses to an open field (OF) test for 10 min at the age of 35 weeks using similar method to Rodenburg et al. [21]. All tested hens were carried to an adjacent separate room, containing a 1.5 × 1.5 m test arena. The testing room was equipped with four LED light lamps, which could be switched according to the hens from different experimental treatments. The arena consisted of four walls (0.8 m high) and a floor made by galvanized iron sheets. In order to prevent unnecessary stress of an individual before the test, all hens were transported from the home cage to the testing arena in a cardboard box and were placed in the middle of the testing arena in darkness. The light was then turned on and the testing person left the room. The experimenter stood behind the door with a viewing window and was not visible to the bird. Measurements taken were the latencies to peep, defecation, walk, the duration of freezing, the number of vocalization and jump.

#### 2.3.2. Avoidance Distance Test, Novel Object Test, Tonic Immobility Test

The procedure for the avoidance distance (AD) test, the novel object (NO) test, and the tonic immobility (TI) test was derived from the Welfare Quality protocol [22] and modified by the previous study by Shi et al. [5]. Fear tests (except for the OF test), were performed at the age of 36 weeks. The AD test was first, and then the NO test. Afterwards, 12 focal hens were selected from each experimental cage for the TI test. For the AD test, the distance from the experimenter’s hand to the front wire mesh of the experimental cage was measured. Six hens were selected from each side of the cage, giving a total of 12 hens per cage. For the NO test, the selected novel object was a plastic stick measuring 60 cm in length with a 3-cm diameter. It was covered with five different colored bands of approximately 2 cm width. The number of hens within 30 cm of the NO was counted every 10 s for a duration of 2 min. Two positions on each side of the treatment cages were chosen (four positions per cage). The number of hens in the four positions in each cage was averaged. The TI test was conducted at the end of the house. For the TI test, the number of inductions and head movements needed and TI duration and latency were recorded for each hen. If the hens were not put into TI after 5 inductions, scores of 0 s for the duration and latency were given to hens, whereas a maximum of 5 was given for the number of inductions. If a hen remained in TI for the maximum testing period of 5 min, a score of 600 s was given for the duration of TI.

### 2.4. Plumage Scores

At 36 weeks of age, after TI test, the plumage coverage condition of 12 focal hens in each experimental cage was individually determined using the three-point scale method described in the Welfare Quality protocol [20] as follows—score 3: no or slight wear, (nearly) complete feathering (only single feathers lacking); score 2: moderate wear, i.e., damaged feathers (worn, deformed) or one or more featherless areas < 5 cm in diameter at the largest extent; score 1: at least one featherless area ≥ 5 cm in diameter at the largest extent. The back, rump, tail, and belly regions of the hen were evaluated. A single score for overall plumage condition was also calculated.

### 2.5. Blood Measurements

All brachial blood samples were collected after fear tests and plumage coverage evaluation over 2 days during week 56. Six focal hens were randomly selected from the marked hens in each experimental cage giving a total number 192 birds. The samples were taken between 14:00 and 17:00 h each day. Blood samples were collected into 2-mL EDTA tubes within 2 min from bird handling to being stored on ice immediately after collection. Then blood samples were sent to Beijing Sino-uk Institute of Biological Technology for basal plasma corticosterone (CORT), thyroxine (T4), and triiodothyronine (T3) analysis, and for whole blood 5-hydroxytryptamine (5-HT) analysis.

### 2.6. Statistical Analysis

Data for each of the individual cage were averaged before analysis, as each experimental cage was treated as a statistical unit. Data were first checked for normality and heterogeneity of variance with and without transformations. Then the statistical analysis was performed using the linear mixed models procedure of SPSS software (IBM SPSS Statistics 22.0, Armonk, NY, USA). Fixed effects included light wavelength and light intensity, while the cage was considered as a random effect. The common model for each parameter contained the two qualitative factors as well as their interactions. Each model was reduced in a stepwise fashion, removing the least significant, highest order interaction in turn until only significant risk factors and interactions remained in the model. Post hoc analyses included pair-wise comparisons between significant factors in order to determine the nature of the significant effects (*p* < 0.05). Pecking frequency and plumage score showed non-normal distributions that were not suitable for transformation, so the Mann–Whitney U test was applied for post hoc group comparisons. Mean comparisons were evaluated on fear responses, cannibalistic injuries and blood parameters by Duncan’s Multiple Range test. Statistical significance was determined at *p* < 0.05 unless otherwise stated.

## 3. Results

### 3.1. Behavioral Observations

The influence of light colors, light intensities and their interaction on the pecking behaviors are shown in Table 2. In comparison with BG, hens in the WL and RL groups had lower frequency of GFP (*p* ≤ 0.004), and no significant difference was found between YO group and other groups for the frequency of GFP. Hens in RL group had the lowest frequency of SFP (*p* ≤ 0.003), whereas hens in the WL group had the highest frequency of SFP (*p* ≤ 0.025), and intermediate frequency of SFP for YO and BG. No significant difference was observed on the SP frequency for all light colors and both light intensities. In addition, hens in the RL and BG groups showed higher ENP activity (*p* ≤ 0.025) than other groups. A significant effect of light intensity on GEP, SFP, and FOP was found. Compared with LLI, HLI showed a higher frequency of GFP (*p* ≤ 0.037), SFP (*p* ≤ 0.023), and FOP (*p* ≤ 0.016). A significant intensity × color interaction was noted for GEP (*p* ≤ 0.001), SFP (*p* ≤ 0.017), ENP (*p* ≤ 0.036), and FOP (*p* ≤ 0.044).

### 3.2. Fear Responses

Table 3 presents the effects of light colors, light intensities and their interaction on the responses of hens to OF tests. No significant differences for light colors were found in the latency to first peep or number of jumps. Hens in the YO group had a significantly longer duration of freezing (*p* ≤ 0.023) than other groups. WL and YO groups had a shorter latency to first defecation (*p* ≤ 0.033) and more vocalizations (*p* ≤ 0.005) than the RL and BG groups. Compared with other groups, hens in the RL group showed a shorter time to first pacing (*p* ≤ 0.044). Hens under HLI had a longer latency to first peep (*p* ≤ 0.003) and a shorter latency to first defecation (*p* ≤ 0.034). A significant intensity × color interaction was noted for the duration of freezing (*p* ≤ 0.042), the latency to first defecation (*p* ≤ 0.022), the latency to first pacing (*p* ≤0.034) and the number of vocalizations (*p* ≤ 0.028).

Table 4 shows the effects of light colors, light intensities and their interaction on the responses of hens to TI tests, NO test, and AD test. Compared with RL and BG, hens in the WL and YO groups had a significantly longer TI duration (*p* ≤ 0.042). More hens in the RL went significantly closer (*p* ≤ 0.026) to the novel object and within a shorter distance (*p* ≤ 0.026) to human compared with WL, YO and BG. Compared with LLI, hens under HLI showed a significant longer TI duration (*p* ≤ 0.011), and within a shorter distance to the human (*p* ≤ 0.042) in AD test. In addition, there was a significant intensity × color interaction for the duration (*p* ≤ 0.041) and latency (*p* ≤ 0.035) of the TI test, and the responses to the NO test (*p* ≤ 0.034), and to the human (*p* ≤ 0.022).

### 3.3. Plumage Evaluation

Table 5 shows the effects of light colors, light intensities and their interaction on the plumage condition of four specific body regions and the overall score of the plumage evaluation. Hens in WL and YO had a lower score for back (*p* ≤ 0.036) compared with RL and BG. Hens in YO had the lowest score for rump (*p* ≤ 0.046) in comparison with other groups. There were no significant differences for the score of tail between light color treatments. For belly region, hens caged in RL and BG groups had a higher score (*p* ≤ 0.037) than WL and YO groups. For overall score, hens in RL and BG were highest (*p* ≤ 0.003), whereas hens in the YO were lowest (*p* ≤ 0.025), and intermediate for WL. Compared with HLI, hens under LLI had a higher score for back region (*p* ≤ 0.022) and a higher overall plumage score (*p* ≤ 0.006). In addition, significant intensity × color interactions were noted for plumage score of all body parts and the overall score (*p* ≤ 0.05).

### 3.4. Blood Parameters, Mortality, and Cannibalistic Injuries

Table 6 shows the effects of light colors, light intensities and their interaction on blood parameters, mortality, and cannibalistic injuries. Hens in RL had a higher concentration of 5-HT (*p* ≤ 0.05) and a lower concentration of CORT (*p* ≤ 0.007) than WL and YO. There was a significant difference between groups for the heterophil to lymphocyte ratio (H/L ratio), with the H/L ratio of hens in YO being the highest (*p* ≤ 0.013), and that of hens in RL being the lowest (*p* ≤ 0.028). The H/L ratio of hens in WL was higher compared with BG (*p* ≤ 0.044). Compared with HLI, hens under LLI had a higher 5-HT concentration (*p* ≤ 0.042) and a lower CORT concentration (*p* ≤ 0.006). The heterophil to lymphocyte ratio (*p* ≤ 0.024) was significantly higher under HLI than LLI. No significant differences were found in the concentration of T3 and T4 between light treatments. Mortality from cannibalism for RL was significantly lower (*p* ≤ 0.026) compared with other groups. Cannibalistic injuries for the light treatments presented a similar trend toward to the mortality from cannibalism. In comparison with HLI, hens under LLI had less cannibalistic injuries (*p* ≤ 0.010), and a lower rate of mortality from cannibalism (*p* ≤ 0.026).

## 4. Discussion

Behavior is a good indicator for the evaluation of laying hen welfare. In this experiment, hens under RL and BG tended to express more frequent GFP and ENP than birds exposed to the other two lighting colors, but a lower SFP frequency under RL (especially compared with WL and YO, with BG being intermediate). These results suggest that hens under RL and BG were more engaged in explorative behavior. We also noted that the pecking activities were promoted by high light intensity and hens under high light intensity were more vulnerable to suffering from SFP. Clearly, pecking behavior may be affected by the wavelength of light as well as by light intensity. Huber-Eicher et al. [16] investigated the effects of colored LED illumination on behavior of laying hens. Hens under green light spent more time on pecking at objects and had more frequent pecking at conspecifics compared with red and white light. Hens under red lighting showed less often severe pecks or distress calls than hens under white light, with green light being intermediate. Mohammed et al. [17] looked at the behavior of laying hens under four different light sources. Higher frequency of GFP and aggressive behavior were increased by blue light and high light intensity. This current study confirm these findings that red light alleviates SFP. The higher contribution of longer wavelengths contained in red light may have reduced SFP behavior, although this needs confirmation. This effect was due to the wavelength and should not be confused with eventual effects of intensity. There is now a general agreement that a particular causative factor that is positively correlated with FP is the inhibition of foraging or dust bating behaviors, such as ground pecking or ENP [23]. It has been suggested that FP is a redirection of oral behavior toward conspecific under barren conditions [24]. In our study, the hens under RL and BG spent more time in their explorative pecking behaviors (GFP and ENP) compared to hens of the other treatments; therefore, attention and severe pecks of the hens shifted from conspecifics towards the surroundings. In other studies, Sultana et al. [7] studied the effect of various LED light color on the behavior of laying hens and indicated that hens in red light were more active and expressed more feather pecking than those of hens in blue light. Prayitno et al. [13] suggested that broilers illuminated with red light showed more aggression and did more floor pecking than birds under white, green, or blue light. Similar increases in aggressive behaviors were recorded in a separate investigation of broilers maintained under red, compared with blue, lighting through to 8 weeks [25]. These results are likely a consequence of the perceived increased intensity, as broilers are more sensitive to this range of the spectrum than that measured by lux [26], and birds have greater visual acuity in red light, while higher light intensity increases aggression. Long wavelengths may alter the reflectance of both the plumage of hens and the appearance of the experimental houses [20,27]. This may well make plumage and objects within the environment more attractive for the birds to peck at and explore. However, Leighton et al. [28] suggested that light sources do not affect these behaviors. Lewis and Morris [29] also mentioned that light color appears to have minimal influence on FP, as red light would reach the hypothalamus more rapidly than blue light. In the above studies that differ from the results in the present experiment, only Sultana et al. used LED light. It is difficult to reach a consistent conclusion from previous studies about wavelength effects upon FP. The discrepancy between the results may be caused by the differences of spectral sensitivity of the fowl, the spectral output of the light sources, the adaptability of birds to particular light environment over time, the housing system, stocking density, group size, and so on. Those aspects complicate direct comparisons of the data. The reduction in SFP under red light needs further evaluation because it could be of interest in commercial production situations.

The results in the present experiment indicated that hens caged in RL had effectively reduced mortality from cannibalism and cannibalistic injuries, in accordance with the finding of Wells [14] who found that the employment of red filters or red paint to light sources may be a simple and effective method in alleviating SFP and cannibalism. However, it may be surprising that in spite of the probable differences in the intensity perceived by hens, even where the light had been adjusted being equated for irradiance, wavelength generally did not significantly affect mortality rates in broilers [30]. The parent-stock hens in colony cages were confined together with roosters. The frequent mounting behavior may generate inferior back and rump plumage conditions, which resulted in hens suffering from injuries or scratches on the back and rump. There is a risk of severe feather pecking and cannibalism, especially if there is hemorrhage, broken skin, and fresh wounds. Therefore, the explanation of the red light reducing mortality from cannibalism and cannibalistic injuries may be that the birds cannot easily see red blood or fresh wounds in red light [31]. The elevated mortality and cannibalistic injuries under high light intensity noted in the present study was in accordance with Kjaer and Vestergaard [9], who suggested that high light intensity in both rearing and laying periods tended to increase mortality during laying, especially due to cannibalism.

Light sources have influences on plumage condition of hens through the influences on FP, as described by Long et al. [18], who showed that different light sources might affect plumage condition as judged by the incidence of feather pecking. In the current study, back, rump, belly, and overall plumage condition of the hens under RL and BG tended to be superior to those under WL and YO. Also, the increased plumage damage under high light intensity found in the current experiment confirms previous findings by Hughes and Duncan [11], Hughes and Black [32], and Allen and Perry [33], who indicated that high light intensity strongly affects the occurrence and severity of FP in laying hens with higher light intensity resulting in more damage. According to Bilcík and Keeling [34], GFP does not contribute to feather damage, while SFP is identified as the major cause of feather pulling, damaging, and plucking. Huber-Eicher and Sebö [35] suggested that at an early-age GFP is prevalent, whereas more SFP can develop later, resulting in more deteriorated plumage in older hens, consistent with our observations. Therefore, it could be speculated that hens under RL and BG had a better plumage condition which may attribute to being engaged less in SFP.

Reactions to humans or a new environment are widely employed to estimate the fearfulness of hens [36]. The ability to deal with this situation reflects the stability of the nervous system and the degree of individual excitability [3]. In this experiment, it seems that the likelihood that hens under RL and BG and caged in low light intensity approaching the NO was higher, the duration of the TI test was shorter, and the distance of the AD test was closer than hens in WL and YO and high light intensity. In addition, WL and YO tended to cause longer freezing time, longer latencies, and more distress calls. This indicated that hens caged in WL and YO were more fearful and susceptive to fear tests. However, the results of the study were in disagreement with those of Scott and Siopes [36], who found that no behavioral indications of stress were observed when mature turkey hens were exposed to blue, green, red, or white illumination of the same photon flux from commercial lamps between 30 and 53 weeks. One possibility for the discrepancy may be that the different breeds of hens may respond to light conditions differently. Studies have shown that the fearfulness of hens was associated with feather damage in commercial breeding [37]. The results are in accordance with those of the study by Johnsen et al. [38], which reported that severely feather-pecked birds tended to have an inferior feather coverage condition and were more fearful than birds with minor pecking damage. Hughes and Duncan [11] also found that fearful behavior was associated with greater feather loss. Other studies suggested that on an individual and flock level, having high levels of fear at a young age can become a risk factor for developing feather pecking as adult [39]. Therefore, the effects of light condition on behavioral response to fear tests of hens might through the effects on FP.

According to previous studies, thyroidal hormones are considered to be physiological indicators of various forms of stress in fowl [40]. Triiodothyronine (T3) regulates the metabolic rate and T4 is considered to be inducing molting of laying hens [41]. However, in this experiment, T3 and T4 concentrations were not affected by the light treatments. The hormones may be correlated with the quality of feather coverage. In addition, the CORT and 5-HT levels have been proven to be associated with fearfulness and feather pecking [42]. Hens caged under RL and low light intensity tended to have a higher concentration of 5-HT, a lower CORT concentration, and a lower ratio of heterophils to lymphocytes than WL and YO, which suggested that hens treated with RL and low light intensity showed a lower stress response. The results of the study were in disagreement with those of Olanrewaju et al. [43]. who found that there were no effects of light sources on plasma CORT concentrations. Scott and Siopes [36] also indicated that blue, green, red, and white lights were not stressful to the birds. However, sampling data for the 45 and 53 week showed that the birds exposed to red light had the lowest proportion of heterophils and the narrowest H/L ratio [36]. However, the effect of light color on the significant effect on CORT concentration and H/L ratio in this study was not clear; this effect might be caused by the effect of light condition on hens’ behavior. As previous studies regarding the effect of light color on fear response of layer hens are scarce, a direct comparison is difficult. However, these results showed a consistent tendency towards greater CORT concentration [3], lower levels of whole blood 5-HT [44], and a higher H/L ratio [44] in highly fearful hens, which showed long tonic immobility durations, a far avoidance distance, and particular fearfulness of novel objects in this study. Cockrem [3] found that corticosterone responses and fearfulness were linked and indicated that greater fearfulness was accompanied by larger corticosterone responses to potentially threatening stimuli. Bolhuis et al. [42] suggested that hens from the generation of the low mortality line showed less fear-related behavior and displayed higher whole-blood 5-HT concentrations. José et al. [44] indicated that hens suffering from cloacal cannibalism were more asymmetrical, stressed, and fearful than non-vent pecked birds, with increased heterophil to lymphocyte ratio and tonic immobility duration. However, in the present study, the differences of the level of fearfulness were not reflected precisely in the concentration of thyroidal hormones. Therefore, measuring the thyroid hormone may not be a particularly appropriate method for evaluating stress in hens, because some factors related to welfare appear to lead to a rise, whereas others result in a fall [40]. Under closely controlled conditions, circulating stress hormones can be a measure of the hen’s reaction to its environment. The condition is apparently not so straightforward in actual operations. The only safe conclusion seems to be that for stress hormones too many uncontrolled factors exert an effect to permit these indicators to be employed as simple and practical assessment of welfare.

## 5. Conclusions

The results of this study illustrate that different light color and light intensity influenced the behavior and fear response of laying hens. Hens treated with RL and low light intensity in natural mating colony cages during the laying period showed a lower frequency of SFP, less damaged plumage, were less fearful, and had lower physiological indicators of stress. In addition, RL could reduce mortality from cannibalism and cannibalistic injuries. Transforming the light color to red or dimming the light could be regarded as an effective method to reduce the risk of FP and cannibalism and alleviate the fear responses of layer breeders in natural mating colony cages. Such knowledge might help to understand FP behavior and stress susceptibility of hens in natural mating colony cages and will provide a basis for the development and optimization of cage equipment and regulation of the light environment.

## Figures and Tables

**Table 1:** Details of measurement samplings.

Measurement Samplings	Details
Pecking behaviors	12 hens/cage, 4 cages/treatment, 48 hens/treatment, at 34 weeks
Open filed test	6 hens/cage, 4 cages /treatment, 24 hens/treatment, at 35 weeks
Tonic immobility test	12 hens/cage, 4 cages /treatment, 48 hens/treatment, at 36 weeks
Avoidance distance test	12 hens/cage, 4 cages /treatment, 48 hens/treatment, at 36 weeks
Nobel object test	4 cages/treatment, at 36 weeks
Plumage condition	12 hens/cage, 4 cages /treatment, 48 hens/treatment, at 36 weeks
Blood parameters	6 hens/cage, 4 cages/treatment, 24 hens/treatment, at 56 weeks
Dead birds	9 cages/treatment, during the experiment
Cannibalistic injuries	12 hens/cage, 4 cages/treatment, 48 hens/treatment, during the experiment

**Table 2:** Means (±SE) of pecking behaviors of hens in response to light colors and light intensities ^*^.

Item	Behaviors ^3^				
	GFP	SFP	SP	ENP	FOP
Light intensity ^1^					
HLI	7.83 ± 0.54 ^a^	4.02 ± 0.28 ^a^	0.56 ± 0.08	11.24 ± 0.92	18.49 ± 2.13 ^a^
LLI	5.87 ± 0.61 ^b^	2.91 ± 0.32 ^b^	0.49 ± 0.07	11.01 ± 1.03	16.76 ± 2.05 ^b^
Light color ^2^					
WL	5.78 ± 0.62 ^b^	3.78 ± 0.33 ^a^	0.51 ± 0.03	8.93 ± 0.88 ^c^	20.17 ± 1.87 ^a^
RL	6.22 ± 0.55 ^b^	0.88 ± 0.12 ^c^	0.58 ± 0.08	14.32 ± 1.75 ^a^	17.20 ± 1.56 ^bc^
YO	7.25 ± 0.63 ^ab^	2.87 ± 0.35 ^ab^	0.56 ± 0.09	9.84 ± 1.02 ^c^	17.07 ± 1.88 ^bc^
BG	7.38 ± 0.68 ^a^	2.31 ± 0.04 ^b^	0.46 ± 0.05	11.41 ± 1.33 ^b^	16.03 ± 1.96 ^c^
Intensity-Color					
WL–HLI	6.94 ± 0.63 ^b^	4.63 ± 0.66 ^a^	0.49 ± 0.05	8.68 ± 1.13 ^c^	22.83 ± 3.01 ^a^
WL–LLI	4.62 ± 0.42 ^c^	2.92 ± 0.18 ^b^	0.52 ± 0.03	9.18 ± 1.02 ^c^	17.51 ± 1.92 ^bc^
RL–HLI	7.35 ± 0.56 ^b^	0.83 ± 0.07 ^c^	0.68 ± 0.05	15.41 ± 1.75 ^a^	18.35 ± 1.87 ^b^
RL–LLI	5.08 ± 0.37 ^c^	0.92 ± 0.09 ^c^	0.47 ± 0.06	13.22 ± 1.24 ^b^	16.04 ± 1.44 ^c^
YO–HLI	8.87 ± 0.89 ^ab^	3.41 ± 0.42 ^b^	0.58 ± 0.06	9.33 ± 0.88 ^c^	16.96 ± 2.11 ^c^
YO–LLI	5.62 ± 0.38 ^c^	2.33 ± 0.21 ^b^	0.54 ± 0.07	10.35 ± 1.65 ^c^	17.17 ± 1.65 ^bc^
BG–HLI	8.14 ± 0.76 ^a^	2.75 ± 0.03 ^b^	0.49 ± 0.04	11.54 ± 1.59 ^bc^	15.81 ± 1.66 ^c^
BG–LLI	6.62 ± 0.64 ^bc^	1.87 ± 0.03 ^bc^	0.42 ± 0.03	11.27 ± 1.73 ^bc^	16.32 ± 2.25 ^c^
Source of variation					
Light intensity	0.004	0.003	0.234	0.316	0.037
Light color	0.001	0.001	0.104	0.006	0.005
Intensity × Color	0.001	0.017	0.742	0.036	0.044

^a–c^ Means within a column and effects that lack common superscripts differ significantly (*p* ≤ 0.05); * Values shown are the pecking frequency (number of pecks per bird/5 min) of four replicate cages with 12 hens per cage; ^1^ Light intensity: HLI = high light intensity, LLI = low light intensity; ^2^ Light colors: WL = white light, RL = red light, YO = yellow-orange light, BG = blue green light; ^3^ Behaviors: GFP = gentle feather pecking, SFP = severe feather pecking, SP = aggressive pecking, ENP = environmental pecking, FOP = food pecking.

**Table 3:** Means (±SE) of responses of hens to the OF test in response to light colors and light intensities *.

Item	OF Tests ^3^					
	Duration of Freezing (s)	Latency to First Peep (s)	Latency to First Defecation (s)	Latency to First Pacing (s)	Number of Vocalizations	Number of Jumps
Light intensity ^1^						
HLI	240.52 ± 21.25	17.44 ± 2.04 ^a^	264.45 ± 34.52 ^b^	6.88 ± 1.87	54.55 ± 7.62	5.78 ± 0.85
LLI	211.23 ± 22.33	13.92 ± 2.04 ^b^	310.17 ± 41.56 ^a^	5.62 ± 1.65	59.68 ± 7.56	5.86 ± 1.02
Light color ^2^						
WL	229.92 ± 25.23 ^b^	14.56 ± 1.95	241.26 ± 35.23 ^b^	8.29 ± 1.15 ^a^	65.67 ± 8.55 ^a^	7.89 ± 1.45
RL	216.57 ± 24.38 ^b^	17.43 ± 1.34	365.64 ± 26.58 ^a^	3.77 ± 1.45 ^b^	34.62 ± 9.67 ^b^	4.37 ± 0.98
YO	254.62 ± 21.16 ^a^	17.84 ± 2.04	211.48 ± 23.14 ^b^	7.96 ± 1.36 ^a^	90.06 ± 11.22 ^a^	7.06 ± 1.04
BG	211.05 ± 27.45 ^b^	12.80 ± 2.15	332.55 ± 35.26 ^a^	6.99 ± 1.47 ^a^	39.81 ± 8.26 ^b^	3.95 ± 0.55
Intensity–Color						
WL–HLI	236.44 ± 27.14 ^b^	17.24 ± 2.25	205.15 ± 43.88 ^b^	10.15 ± 2.33 ^a^	56.24 ± 12.56 ^bc^	6.88 ± 1.62
WL–LLI	209.40 ± 23.44 ^b^	11.88 ± 2.14	277.37 ± 48.36 ^ab^	6.42 ± 1.78 ^bc^	75.10 ± 18.36 ^ab^	8.90 ± 1.56
RL–HLI	227.80 ± 21.71 ^b^	20.18 ± 2.36	357.45 ± 38.74 ^a^	3.25 ± 1.78 ^c^	44.78 ± 8.95 ^cd^	5.10 ± 1.56
RL–LLI	205.33 ± 24.56 ^b^	14.68 ± 2.14	373.83 ± 32.35 ^a^	4.28 ± 1.78 ^c^	24.45 ± 6.25 ^d^	3.64 ± 1.33
YO–HLI	276.23 ± 27.14 ^a^	18.94 ± 3.24	166.60 ± 63.24 ^c^	8.36 ± 2.33 ^ab^	83.33 ± 19.33 ^ab^	6.74 ± 1.33
YO–LLI	233.64 ± 26.38 ^b^	16.74 ± 2.25	256.35 ± 33.64 ^b^	7.55 ± 2.02 ^ab^	96.80 ± 20.14 ^a^	7.38 ± 2.02
BG–HLI	223.35 ± 28.24 ^b^	13.40 ± 2.36	328.37 ± 35.56 ^a^	5.76 ± 1.13 ^bc^	36.62 ± 6.55 ^cd^	4.40 ± 0.64
BG–LLI	198.83 ± 30.15 ^b^	12.36 ± 1.98	336.46 ± 41.37 ^a^	4.23 ± 2.14 ^c^	43.28 ± 7.69 ^cd^	3.50 ± 0.53
Source of variation						
Light intensity	0.164	0.003	0.034	0.416	0.057	0.753
Light color	0.021	0.501	0.004	0.016	0.005	0.175
Intensity–Color	0.042	0.717	0.022	0.034	0.028	0.864

^a–c^ Means within a column and effects that lack common superscripts differ significantly (*p* ≤ 0.05); * Values shown are the responses of hens to OF test of four replicate cages with six hens per cage; ^1^ Light intensity: 1: HLI = high light intensity, LLI = low light intensity; ^2^ Light colors: WL = white light, RL = red light, YO = yellow-orange light, BG = blue green light; ^3^ OF test = open field test.

**Table 4:** Means (±SE) of responses of hens to the TI test, NO test, and AD test in response to light colors and light intensities *.

Item	TI Tests ^3^				NO Test ^4^	AD Test ^5^
	Duration (s)	Latency (s)	Induction (no)	HM (no)	Number of hens	Distance (cm)
Light intensity ^1^						
HLI	109.77 ± 3.43 ^a^	20.67 ± 1.02	2.65 ± 0.08	4.72 ± 0.23	12.78 ± 0.37	23.92 ± 0.42 ^a^
LLI	95.23 ± 4.86 ^b^	19.31 ± 0.88	2.52 ± 0.11	4.80 ± 0.23	13.66 ± 0.28	18.38 ± 0.61 ^b^
Light color ^2^						
WL	118.86 ± 3.44 ^a^	19.21 ± 1.45	2.54 ± 0.11	5.43 ± 0.22	11.66 ± 0.23 ^b^	25.20 ± 0.47 ^a^
RL	85.43 ± 4.01 ^b^	17.67 ± 2.01	2.47 ± 0.17	4.21 ± 0.24	14.85 ± 0.23 ^a^	22.27 ± 0.56 ^ab^
YO	110.39 ± 3.05 ^a^	21.93 ± 1.88	2.79 ± 0.15	4.31 ± 0.31	12.96 ± 0.32 ^b^	24.15 ± 0.81 ^a^
BG	93.11 ± 4.64 ^b^	21.17 ± 2.75	2.54 ± 0.15	5.09 ± 0.24	13.42 ± 0.31 ^b^	18.08 ± 0.33 ^b^
Intensity–Color						
WL–HLI	125.36 ± 3.87 ^a^	20.35 ± 1.05 ^ab^	2.65 ± 0.14	5.02 ± 0.41	12.24 ± 0.31 ^b^	28.83 ± 0.56 ^a^
WL–LLI	112.35 ± 3.73 ^ab^	18.07 ± 1.05 ^bc^	2.42 ± 0.11	5.83 ± 0.34	11.07 ± 0.29 ^b^	21.57 ± 0.59 ^b^
RL–HLI	90.27 ± 5.45 ^cd^	16.17 ± 1.88 ^c^	2.75 ± 0.13	4.25 ± 0.48	13.88 ± 0.35 ^ab^	29.35 ± 0.81 ^a^
RL–LLI	85.58 ± 4.85 ^d^	19.16 ± 2.02 ^abc^	2.18 ± 0.15	4.17 ± 0.35	15.82 ± 0.28 ^a^	15.18 ± 0.62 ^c^
YO–HLI	118.33 ± 5.75 ^a^	23.67 ± 2.04 ^a^	2.75 ± 0.15	4.68 ± 0.29	13.75 ± 0.27 ^ab^	27.77 ± 0.67 ^a^
YO–LLI	102.44 ± 5.32 ^bc^	20.18 ± 2.46 ^ab^	2.83 ± 0.12	3.93 ± 0.52	12.16 ± 0.31 ^b^	20.52 ± 0.73 ^b^
BG–HLI	105.13 ± 3.24 ^b^	22.50 ± 3.48 ^a^	2.44 ± 0.15	4.92 ± 0.32	11.25 ± 0.30 ^b^	19.92 ± 0.66 ^bc^
BG–LLI	81.08 ± 3.09^d^	19.83 ± 1.73 ^ab^	2.64 ± 0.15	5.25 ± 0.28	15.58 ± 0.33 ^a^	16.23 ± 0.72 ^bc^
Source of variation						
Light intensity	0.003	0.244	0.739	0.243	0.684	0.004
Light color	0.034	0.612	0.832	0.252	0.029	0.006
Intensity–Color	0.041	0.035	0.466	0.715	0.034	0.022

^a–d^ Means within a column and effects that lack common superscripts differ significantly (*p* ≤ 0.05); * Values shown are the responses of hens to the TI test, AD test of four replicate cages with 12 hens per cage, and the responses to the NO test of four replicate cages; ^1^ Light intensity: HLI = high light intensity, LLI = low light intensity; ^2^ Light colors: WL = white light, RL = red light, YO = yellow-orange light, BG = blue green light; ^3^ TI test = tonic immobility test, HM = head movement; ^4^ NO test = novel object test; ^5^ AD test = avoidance distance test.

**Table 5:** Means (±SE) of plumage score of hens in response to light colors and light intensities *.

Item	Body Part				
	Back	Rump	Tail	Belly	Overall
Light intensity ^1^					
HLI	2.54 ± 0.17 ^b^	2.32 ± 0.22	2.67 ± 0.15	2.76 ± 0.22	2.52 ± 0.13 ^b^
LLI	2.72 ± 0.17 ^a^	2.45 ± 0.21	2.87 ± 0.18	2.71 ± 0.23	2.76 ± 0.15 ^a^
Light color ^2^					
WL	2.58 ± 0.15 ^b^	2.41 ± 0.21 ^a^	2.71 ± 0.21	2.57 ± 0.22 ^b^	2.45 ± 0.20 ^b^
RL	2.85 ± 0.15 ^a^	2.50 ± 0.21 ^a^	2.85 ± 0.24	2.94 ± 0.14 ^a^	2.81 ± 0.23 ^a^
YO	2.52 ± 0.17 ^b^	2.17 ± 0.17 ^b^	2.73 ± 0.24	2.54 ± 0.19 ^b^	2.20 ± 0.23 ^c^
BG	2.72 ± 0.13 ^a^	2.47 ± 0.18 ^a^	2.80 ± 0.19	2.91 ± 0.21 ^a^	2.71 ± 0.18 ^a^
Intensity–Color					
WL–HLI	2.63 ± 0.17 ^a^	2.37 ± 0.24 ^b^	2.58 ± 0.23 ^b^	2.58 ± 0.18 ^b^	2.43 ± 0.18 ^b^
WL–LLI	2.52 ± 0.15 ^b^	2.44 ± 0.21 ^ab^	2.83 ± 0.24 ^a^	2.55 ± 0.19 ^b^	2.47 ± 0.15 ^b^
RL–HLI	2.81 ± 0.15 ^a^	2.42 ± 0.19 ^ab^	2.77 ± 0.19 ^ab^	2.92 ± 0.20 ^a^	2.77 ± 0.16 ^a^
RL–LLI	2.89 ± 0.14 ^a^	2.58 ± 0.24 ^a^	2.92 ± 0.26 ^a^	2.96 ± 0.18 ^a^	2.84 ± 0.14 ^a^
YO–HLI	2.44 ± 0.18 ^b^	2.08 ± 0.23 ^c^	2.59 ± 0.23 ^b^	2.58 ± 0.24 ^b^	2.17 ± 0.16 ^b^
YO–LLI	2.73 ± 0.15 ^a^	2.25 ± 0.24 ^bc^	2.88 ± 0.24 ^a^	2.49 ± 0.22 ^b^	2.42 ± 0.15 ^b^
BG–HLI	2.67 ± 0.14 ^a^	2.41 ± 0.20 ^ab^	2.75 ± 0.22 ^ab^	2.96 ± 0.24 ^a^	2.70 ± 0.20 ^a^
BG–LLI	2.73 ± 0.13 ^a^	2.52 ± 0.23 ^ab^	2.85 ± 0.24 ^a^	2.85 ± 0.14 ^a^	2.72 ± 0.16 ^a^
Source of variation					
Light intensity	0.001	0.474	0.524	0.175	0.024
Light color	0.01	0.012	0.132	0.014	0.019
Intensity–Color	0.01	0.005	0.024	0.036	0.004

^a–c^ Means within a column and effects that lack common superscripts differ significantly (*p* ≤ 0.05); * Values shown are the plumage score of four replicate cages with 12 hens per cage; ^1^ Light intensity: 1: HLI = high light intensity, LLI = low light intensity; ^2^ Light colors: WL = white light, RL = red light, YO = yellow-orange light, BG = blue green light.

**Table 6:** Means (±SE) of blood parameters, mortality and cannibalistic injuries of hens in response to light colors and light intensities *.

Item	Blood Parameters ^3^					Mortality and Injuries ^4^	
	T3 (ng/mL)	T4 (ng/mL)	5-HT (ng/mL)	CORT (ng/mL)	H/L	Cannibalism (%)	Injuries (*n*)
Light intensity ^1^							
HLI	0.55 ± 0.01	15.10 ± 0.32	28.11 ± 1.22 ^b^	5.19 ± 0.15 ^a^	0.50 ± 0.01^a^	5.88 ± 0.32 ^a^	0.48 ± 0.44 ^a^
LLI	0.60 ± 0.01	15.75 ± 0.24	30.64 ± 0.93 ^a^	4.66 ± 0.31 ^b^	0.41 ± 0.01 ^b^	4.44 ± 0.58 ^b^	0.28 ± 0.36 ^b^
Light color ^2^							
WL	0.65 ± 0.01	16.76 ± 0.28	27.04 ± 1.22 ^b^	4.90 ± 0.55 ^a^	0.49 ± 0.01 ^b^	5.31 ± 0.57 ^a^	0.40 ± 0.02 ^a^
RL	0.49 ± 0.02	14.04 ± 0.44	30.73 ± 0.89 ^a^	4.06 ± 0.25 ^b^	0.32 ± 0.01 ^d^	2.65 ± 0.33 ^c^	0.18 ± 0.01 ^c^
YO	0.56 ± 0.01	15.05 ± 0.32	28.14 ± 2.13 ^b^	5.08 ± 0.27 ^a^	0.60 ± 0.01 ^a^	6.06 ± 0.46 ^a^	0.46 ± 0.04 ^a^
BG	0.60 ± 0.02	15.85 ± 0.17	30.61 ± 1.07 ^a^	4.52 ± 0.33 ^ab^	0.41 ± 0.01 ^c^	4.63 ± 0.32 ^b^	0.29 ± 0.04 ^b^
Intensity–Color							
WL–HLI	0.62 ± 0.01	16.48 ± 0.40	26.10 ± 4.61 ^c^	4.96 ± 0.62 ^a^	0.53 ± 0.01 ^b^	5.93 ± 0.72 ^a^	0.51 ± 0.04 ^a^
WL–LLI	0.67 ± 0.01	17.03 ± 0.29	27.98 ± 1.77 ^bc^	4.84 ± 0.18a	0.44 ± 0.01 ^c^	4.69 ± 0.65 ^b^	0.29 ± 0.04 ^b^
RL–HLI	0.45 ± 0.01	13.71 ± 0.44	28.81 ± 0.84 ^b^	5.63 ± 0.30 ^a^	0.35 ± 0.01 ^d^	2.46 ± 0.30 ^c^	0.19 ± 0.03 ^c^
RL–LLI	0.53 ± 0.02	14.36 ± 0.38	32.64 ± 0.88 ^a^	4.08 ± 0.33 ^b^	0.28 ± 0.01 ^e^	2.83 ± 0.33 ^c^	0.17 ± 0.02 ^c^
YO–HLI	0.52 ± 0.02	14.66 ± 0.22	28.51 ± 0.89 ^b^	5.04 ± 0.21 ^a^	0.65 ± 0.01 ^a^	6.30 ± 0.85 ^a^	0.49 ± 0.04 ^a^
YO–LLI	0.59 ± 0.02	15.44 ± 0.21	31.76 ± 0.83 ^a^	5.11 ± 0.39 ^a^	0.55 ± 0.01 ^b^	5.81 ± 0.72 ^a^	0.42 ± 0.04 ^a^
BG–HLI	0.59 ± 0.02	15.53 ± 0.17	29.03 ± 1.48 ^b^	5.11 ± 0.52 ^a^	0.45 ± 0.01 ^c^	4.81 ± 0.60 ^b^	0.32 ± 0.03 ^b^
BG–LLI	0.61 ± 0.02	16.16 ± 0.20	30.19 ± 0.91 ^b^	4.60 ± 0.39 ^b^	0.37 ± 0.01 ^d^	4.44 ± 0.54 ^b^	0.25 ± 0.02 ^b^
Source of variation							
Light intensity	0.431	0.579	0.042	0.001	0.041	0.010	0.005
Light color	0.643	0.412	0.021	0.003	0.037	0.010	0.007
Intensity–Color	0.233	0.283	0.018	0.001	0.008	0.001	0.003

^a–e^ Means within a column and effects that lack common superscripts differ significantly (*p* ≤ 0.05);* Values shown are the plumage score of four replicate cages with 12 hens per cage and the mortality and cannibalistic injuries of four replicate cages; ^1^ Light intensity: 1: HLI = high light intensity, LLI = low light intensity; ^2^ Light colors: WL = white light, RL = red light, YO = yellow-orange light, BG = blue green light; ^3^ Blood parameters: T3 = triiodothyronine, T4 = thyroxine, CORT = corticosterone, 5-HT = serotonin; H/L = the ratio of heterophil to lymphocyte. ^4^ Cannibalism = mortality from cannibalism, Injuries = cannibalistic injuries.
